# Early acetabular cup migration is associated with long-term aseptic loosening: investigation of 1-year and 2-year radiostereometric analysis cohort migration thresholds against acetabular cup survivorship from two national registries

**DOI:** 10.2340/17453674.2026.45784

**Published:** 2026-05-29

**Authors:** Chan Hee CHO, John M ABRAHAMS, Lucian B SOLOMON, Thomas S ROBERTSON, Christopher J WALL, Carl HOLDER, Liza N VAN STEENBERGEN, Bart G PIJLS, Stuart A CALLARY

**Affiliations:** 1 Discipline of Orthopaedics and Trauma, School of Medicine, Adelaide University, Adelaide, SA, Australia; 2 Department of Orthopaedics and Trauma, Royal Adelaide Hospital, Adelaide, SA, Australia; 3 Department of Orthopaedics, Leiden University Medical Centre, Leiden, Netherlands; 4 Australian Orthopaedic Association National Joint Replacement Registry, Adelaide, SA, Australia; 5 Dutch Arthroplasty Register (LROI), Leiden, Netherlands; 6 University of Queensland Rural Clinical School, Queensland, Australia; 7 South Australian Health and Medical Research Institute (SAHMRI), Adelaide, South Australia

## Abstract

**Background and purpose:**

2-year proximal migration thresholds for acetabular cups used at primary total hip arthroplasty were first established in 2012 by matching radiostereometric analysis (RSA) measurements of cup migration with long-term survivorship from cohort studies. Subsequently, there have been new RSA studies reporting migration of contemporary cup designs whose survivorships are available from long-term registry data. The primary aim of this study was to re-evaluate the 2-year postoperative RSA migration thresholds and investigate whether thresholds at time points earlier than 2 years were possible.

**Methods:**

The mean 6-month, 1-, and 2-year proximal migration of each RSA cohort was matched to the pooled 10-year proportion of acetabular cup revision due to aseptic loosening in the Australian Orthopaedic Association National Joint Replacement Registry and the Dutch Arthroplasty Registry in 2023. Revision rates were pooled according to these classifications using a random effects model.

**Results:**

164 implant-survival combinations (228,053 cups), involving 27 cup designs, were identified with RSA migration data to 2 years. At 1 year, mean cup migration of ≤ 0.1 mm was considered acceptable, while migration > 0.8 mm was unacceptable. The pooled proportions of acetabular cup revision in the acceptable, at-risk, and unacceptable threshold categories were 0.36% (95% confidence interval [CI] 0.26–0.47), 1.05% (CI 0.80–1.31), and 5.07% (CI 1.41–8.73) respectively. At 2 years, mean cup migration ≤ 0.2 mm was considered acceptable while mean migration > 1.0 mm had unacceptable survivorship. The pooled proportions of acetabular cup revision in the acceptable, at-risk, and unacceptable threshold categories were 0.55% (CI 0.48–0.62), 1.27% (CI 0.87–1.67), and 21.6% (CI 0.0–54.7), respectively, meaning that acetabular cups with high migration can be identified at both 1 and 2 years.

**Conclusion:**

This study confirmed the existing 2-year migration thresholds and established a new threshold at 1 year. We found no association between continuous migration between 1 and 2 years and 10-year revision rates. The 1-year migration threshold is the earliest surrogate measure for assessing late loosening of the acetabular cup and may identify underperforming implants.

The gold standard for assessing the clinical outcomes of acetabular cup designs in total hip arthroplasty (THA) is long-term registry data, which evaluates implant revision rates at 10 years or longer follow-up [1-3]. A revision rate of an acetabular cup is considered acceptable if it is below 5% at 10 years [4]. Unfortunately, long-term registry survivorship reports rely on an extended follow-up period, during which patients may receive a poorly performing implant [1]. Assessing acetabular cup performance earlier with a validated surrogate method is essential to facilitate safer implementation of new designs in a stepwise approach [2,5].

Radiostereometric analysis (RSA) is used to assess the migration of acetabular cups [6]. The high accuracy of RSA measurements allows assessment of cup designs with relatively small patient cohorts [6,7]. A systematic review by Pijls et al. found acetabular cups with a mean cohort proximal migration > 1.0 mm at 2 years postoperatively were considered to have unacceptable revision rates at 10 years [4]. Acetabular cup migration measured with RSA at 2 years has been used as an early surrogate method to identify poorly performing designs.

During the last decade, since the publication of the migration thresholds [4] the number of RSA studies reporting migration of contemporary cup designs has increased [8]. It is now possible to match cup migration with long-term national registry revision rates for aseptic loosening. The primary aim of this study was to re-evaluate the 2-year postoperative RSA migration thresholds and investigate whether thresholds at time points earlier than 2 years are possible. The secondary aim was to investigate whether a continuous migration threshold between 1 and 2 years was possible to identify whether early continuous migration was associated with long-term loosening.

## Methods

### Study design and data

A recent systematic review by Cho et al. was used to identify acetabular cup designs whose migration after a primary THA had been studied with RSA [8]. From the review, proximal migration, acetabular cup names, and fixation methods were extracted. Based on this information, requests were sent to 2 national joint replacement registries, the Dutch Arthroplasty Register (LROI) [9] and the Australian Orthopaedic Association National Joint Replacement Registry (AOANJRR) [10], to obtain 10-year revision rates due to aseptic loosening for the identified implants, with registry data collected in 2023.

This study is reported according to the STROBE guidelines.

### Categorization of migration thresholds

The thresholds for acetabular cup migration were divided into 3 groups: acceptable, at-risk, and unacceptable. This categorization was based on the previous RSA thresholds of acetabular cup migration by Pijls et al. [4]. Implant-survival combination was defined as a cohort of an acetabular cup design with published RSA migration matched with 10-year revision rate data due to aseptic loosening. The cohort migration of the acetabular cup design was classified as “acceptable“ if all implant-survival combinations had a 10-year revision rate due to aseptic loosening below 5%. The cohort migration of the acetabular cup design was classified as “unacceptable” if all implant-survival combinations had a 10-year revision rate due to aseptic loosening above 5%. The cohort migration of acetabular cup design was categorized as “at-risk” if the implant-survival combinations had 10-year revision rates both lower and higher than 5%.

### Data analysis and synthesis

Only the proximal migration of acetabular cup design was analyzed as it is the most common axis reported [8] and it has previously been used to predict loosening of acetabular cup designs. To evaluate the 2-year proximal migration threshold, the 10-year revision rate for aseptic loosening was matched to the cohort migration of 2-year acetabular cup designs measured with RSA according to implant name and fixation method across both registries. Additionally, all the implant-survival combinations from the previously published study [4] were included in the new analysis. There were multiple implant-survival combinations identified due to multiple RSA measurements and/or registry survivorship reported for the same cup design. For example, if there were 2 RSA studies with the same implant design that had a matching 10-year revision rate from both AOANJRR and LROI there would be 4 total implant-survival combinations (2 x 2 = 4 implant-survival combinations) (Table 1). As previous RSA studies have demonstrated no difference in the early migration patterns between cemented and uncemented acetabular cup designs [11], separate migration thresholds for cemented and uncemented designs were not identified.

**Table 1:** Acetabular cup designs identified from systematic review and matched with registry survivorship data

	Acetabular cup design	Fixation	Coating	No. of RSA cohorts	AOANJRR survivorship data	LROI survivorship data	No. of registry combinations	No. of combinations
1	ABG II	Uncemented	HA coated	2	Yes	Yes	2	4
2	Avantage Reload	Uncemented	HA coated	1	No	Yes	1	1
3	Charnley Flanged	Cemented	NA	8	No	Yes	1	8
4	Delta-TT	Uncemented	Not specified	2	No	Yes	1	2
5	EP-Fit Plus	Uncemented	HA coated	1	Yes	Yes	2	2
			Porous coated	1	Yes	Yes	2	2
6	Exceed ABT	Cemented	NA	4	No	Yes	1	4
	Exceed ABT	Uncemented	Not specified	1	Yes	Yes	2	2
			HA coated	1	Yes	No	1	1
7	Marathon	Cemented	NA	1	No	Yes	1	2
8	Omnifit	Uncemented	HA coated	2	No	Yes	1	2
9	Pinnacle	Uncemented	Porous coated	1	No	Yes	1	2
10	R3 Recap Mangum	Uncemented	Porous coated	1	Yes	Yes	2	2
11	Shells	Uncemented	HA coated	2	No	Yes	1	2
12	Reflection	Cemented	NA	4	Yes	Yes	2	8
			NA	1	Yes	No		1
	Reflection	Uncemented	Porous coated	8	No	Yes	1	8
13	Regenerex	Uncemented	Porous coated	2	Yes	No	1	2
14	RM	Uncemented	Not specified	1	Yes	Yes	2	2
			Porous coated	1	Yes	No	1	1
15	Top Cup	Uncemented	Not specified	1	Yes	Yes	2	2
			HA coated	1	Yes	No	1	1
16	Trabecular Metal	Uncemented	Porous coated	1	Yes	Yes	2	2
17	Trilogy	Uncemented	Porous coated	3	Yes	Yes	2	6
			Porous coated					
			with screws	1	Yes	No	1	1
Total								70

NA = not applicable; AOANJRR = Australian Orthopaedic Arthroplasty National Joint Replacement Registry;

LROI = Dutch Orthopaedic Register.

To investigate new migration thresholds earlier than the current 2-year benchmark, 6-month and 1-year implant-survival combinations were created. Implant-survival combinations were created by matching the aseptic loosening 10-year revision rate data from registry with the 1-year acetabular cup migration measured from RSA studies. Additionally, the 1-year implant-survival combinations using data from the previous study [4] were included in the new analysis. This data was previously unpublished as there were not enough 1-year combinations in 2012. To investigate the continuous migration threshold, the difference in mean migration between 1 and 2 years for each cohort was calculated and then matched with the respective 10-year registry revision rate due to aseptic loosening.

Once the thresholds were established, a random-effects model was used to pool the 10-year proportion of acetabular cup revision due to aseptic loosening by migration category. The estimates are presented as pooled 10-year revision proportion (%) with their corresponding 95% confidence intervals (CIs). Metafor package (version 3.4-0) in R-Studio (R Foundation for Statistical Computing, Vienna, Austria) was used to perform the analysis.

### Ethics, registration, data sharing, use of AI tools, funding, and disclosure

No ethical approval was required for this study as the data was retrieved from previous published studies. This study did not have any prior registrations. The study was not funded by any external or internal party. No AI-assisted tools were used in the preparation or submission of this work. SC held a Research Fellowship from the Hospital Research Foundation during the period of this study. There are no conflicts of interest for any of the authors. Complete disclosure of interest forms according to ICMJE are available on the article page, doi: 10.2340/17453674.2026.45784

## Results

### Investigation of the 2-year migration thresholds

**Figure 1:**
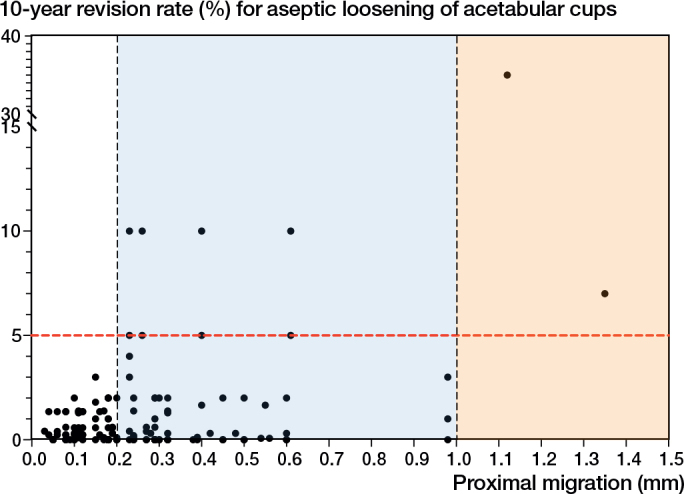
Scatterplot of 2-year mean proximal migration of the acetabular cup determined using RSA (x-axis) and 10-year revision rate of the acetabular cup due to aseptic loosening (y-axis). “Acceptable” mean proximal migration was defined as migration ≤ 0.2 mm. “At-risk” mean proximal migration was defined as migration > 0.2 mm and < 1.0 mm. “Unacceptable” mean proximal migration was defined as migration ≥ 1.0 mm. The black dots represent the 164 implant-survival combinations. The red line represents the 5% revision threshold at 10 years.

The 2012 systematic review reported 94 implant-survival combinations at 2 years, involving 13 acetabular cup designs. In the latest review of published 2-year RSA migration results, there were 70 new implant-survival combinations across 17 acetabular cup designs, 3 of which had also been included in the review [4]. These 2-year RSA migration results were matched to 10-year aseptic loosening revision rates from either the AOANJRR and/or LROI (see Table 1). 164 implant-survival combinations (27 designs) were included in the analysis. Of the 164 implant survival combinations, 86 had migrated ≤ 0.2 mm at 2 years and these 86 had revision rates < 5% at 10 years. There were 76 implant-survival combinations that had migrated > 0.2 mm but < 1.0 mm at 2 years and were classified to be “at risk” of later loosening (Figure 1). There were only 2 implant-survival combinations that had “unacceptable” migration ≥ 1.0 mm at 2 years, and both had revision rates > 5% at 10 years. The pooled proportions of acetabular cup revision in the acceptable, at-risk, and unacceptable threshold categories were 0.55% (CI 0.48–0.62), 1.27% (CI 0.87–1.67), and 21.6% (CI 0.0–54.7), respectively (see Table 3).

**Table 2:** Acetabular cup designs identified from Pijls et al. [4] and matched with survival studies

	Acetabular cup design	Fixation	Coating	No. of RSA cohorts	No. of survival studies	No. of combinations
1	ABG I	Uncemented	HA coated	1	8	8
2	BHR	Uncemented	HA coated	1	4	4
3	Exeter	Cemented	NA	2	3	6
4	Harris-Galante I	Uncemented	Porous coated	2	14	28
5	Harris-Galante II	Uncemented	Porous coated	1	7	7
6	Link V	Uncemented	Threaded	1	1	1
7	Omnifit	Uncemented	HA coated	2	1	2
8	Scanhip	Cemented	NA	1	3	3
9	Wagner Cup	Cemented	NA	1	1	1
10	Charnley Flanged	Cemented	NA	8	3	24
11	Spectron	Cemented	NA	1	1	1
12	Lubinus	Cemented	NA	4	2	8
13	Reflection	Cemented	NA	1	1	1
Total						94

NA = Not applicable

**Table 3:** Pooled 10-year proportion of acetabular cup revision due to aseptic loosening by migration threshold category

Category	Pooled 10-year revision proportion (%) (CI)
2-year proximal migration threshold	
Acceptable	0.55 (0.48–0.62)
At-risk	1.27 (0.87–1.67)
Unacceptable	21.6 (0.0–54.7)
1-year proximal migration threshold	
Acceptable	0.36 (0.26–0.47)
At-risk	1.05 (0.80–1.31)
Unacceptable	5.07 (1.41–8.73)
Continuous migration threshold	
< 0.1mm	0.91 (0.69–1.14)
≥ 0.1mm	2.48 (0.71–4.25)

CI = 95% confidence interval.

### Investigation of earlier proximal migration threshold

**Figure 2:**
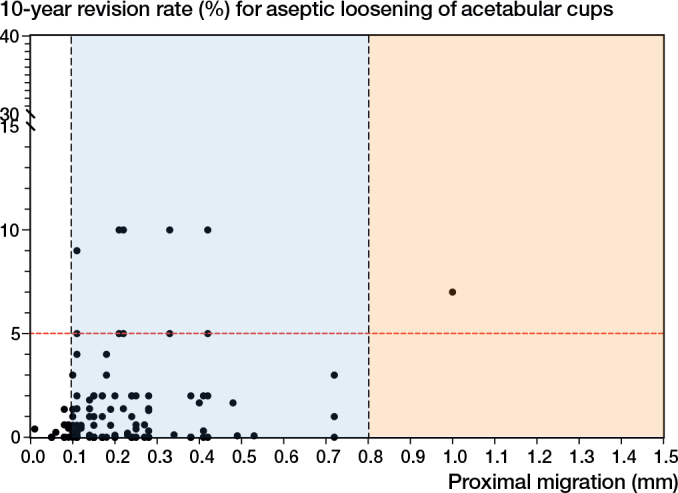
Scatterplot of 1-year mean proximal migration of the acetabular cup determined using RSA (x-axis) and 10-year revision rate of the acetabular cup due to aseptic loosening (y-axis). “Acceptable” mean proximal migration was defined as migration ≤ 0.1 mm. “At-risk” mean proximal migration was defined as migration > 0.1 mm and < 0.8 mm. “Unacceptable” mean proximal migration was defined as migration ≥ 0.8 mm. The black dots represent the 140 implant-survival combinations. The red line represents the 5% revision threshold at 10 years.

There were 93 implant-survival combinations at 1 year, involving 12 acetabular cup designs, (Table 2). 47 new implant-survival combinations, involving 17 acetabular cup designs, 2 of which were identified in the review [4], were identified in the latest review of published 1-year RSA migration results. The 1-year RSA migration results were matched to 10-year aseptic loosening revision rates from either the AOANJRR or LROI. 140 implant-survival combinations (27 designs) were included in the analysis. There were 21 implant-survival combinations that migrated ≤ 0.1 mm at 1 year and were considered to have acceptable migration. These 21 implant-survival combinations all had a revision rate of < 5% at 10 years. There were 118 implant-survival combinations that migrated > 0.1 mm but < 0.8 mm at 1 year and were considered to be “at risk” of later loosening. There was only 1 combination that migrated ≥ 0.8 mm and had a 10-year revision rate of 7%, which was defined as unacceptable (Figure 2). Although 6-month data was initially explored, the number of implant-survivorship combinations available at this time point was too limited to allow meaningful analysis and therefore these were not included in the results. The pooled proportions of acetabular cup revision in the acceptable, at-risk, and unacceptable threshold categories were 0.36% (CI 0.26–0.47), 1.05% (CI 0.80–1.31), and 5.1% (CI 1.4–8.7) respectively (Table 3).

### Investigation of continuous proximal migration threshold between 1 and 2 years

**Figure 3:**
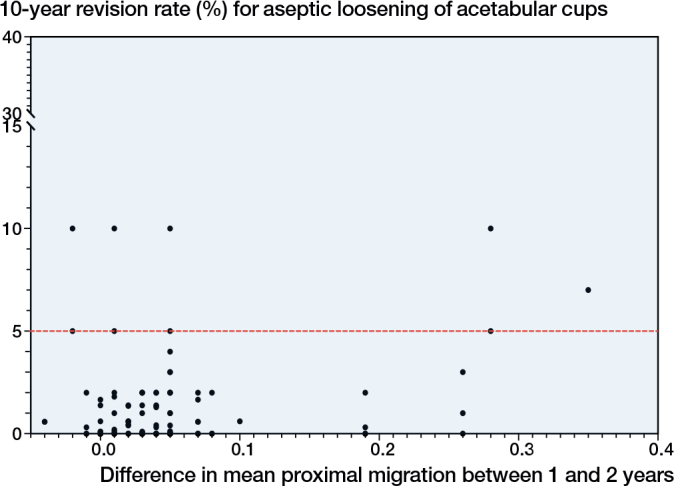
Scatterplot of the difference in mean proximal migration between 1 and 2 years (x-axis) and 10-year revision rate of the acetabular cup due to aseptic loosening (y-axis). The black dots represent the 126 implant-survival combinations. The red line indicates the 5% revision threshold at 10 years.

There were 126 implant-survival combinations. There was no association between continuous migration and 10-year revision rates. There were 8 implant-survival combinations with all-cause revision rates > 5% revision rate benchmark at 10 years (Figure 3). The pooled proportions of acetabular cup revision in the < 0.1 mm and ≥ 0.1 mm threshold categories were 0.91% (CI 0.69–1.14) and 2.48% (CI 0.71–4.25), respectively.

## Discussion

For the first time, it is established that acetabular cups at risk of long-term loosening can be identified using 1-year RSA proximal migration data. We evaluated 2-year proximal migration thresholds and established a new 1-year proximal migration threshold for acetabular cups using published RSA study data matched with long-term revision rates from two national joint replacement registries. The findings of this study support the 2-year threshold being defined as acceptable when acetabular cups migrate ≤ 0.2 mm proximally, at risk when acetabular cups migrate > 0.2 mm but < 1.0 mm proximally, and unacceptable when acetabular cups ≥ 1.0 mm proximally [4]. The addition of 70 new implant-survival combinations strengthened the limitations of the previous thresholds, as they now include modern acetabular cup designs. At 1 year, acetabular cups that migrated ≤ 0.1mm were defined as acceptable. Acetabular cups that migrated proximally > 0.1 mm but < 0.8 mm were classified as at risk of later loosening. Acetabular cups that migrated proximally ≥ 0.8 mm were found to have unacceptable long-term revision rates. The pooled estimates show an increase in 10-year revision proportions with increased early migration across both 1-year and 2-year proximal migration thresholds. There was no association between early continuous RSA migration of acetabular cups between 1 and 2 years and long-term revision rates.

A systematic review by Cho et al. [8] demonstrated that the majority of acetabular cup migration occurs within the first 6 months following primary THA. There were not enough implant-survival combinations with 6-month RSA migration data and matching 10-year registry revision rates. There was sufficient data to establish a 1-year threshold, which allows a shorter clinical study period to assess acetabular cup performance. This is important for both manufacturers and international regulatory bodies, as the orthopedic community moves towards a mandated process of clinical evidence for implants before they are introduced to the market [11]. It is important to note that this is not the sole approach to screening new prostheses. The authors propose that this threshold should be used in conjunction with other surveillance mechanisms capturing patient outcomes, implant-related complications, and/or survivorship. Migration thresholds represent one component of a comprehensive screening strategy and can be used as a surrogate marker for evaluating implant performance of both knee and hip arthroplasty [3,12]. Additionally, computed tomography based radiostereometric analysis (CT-RSA) is a validated alternative method to conventional RSA for measuring implant migration [13]. CT-RSA addresses limitations of traditional RSA, including the need for intraoperative tantalum bead insertion and prospective radiographic examinations above a calibration cage. CT-RSA has been shown to have accuracy and precision comparable to conventional RSA [13] and, therefore, these thresholds are expected to remain applicable for CT-RSA studies.

Continuous migration is the ongoing movement of an implant. It has been theorized that excessive early continuous migration of orthopedic implants would be an indicator of long-term failure as it may indicate poor osseointegration or stability postoperatively [14-16]. Our study is the first to explore continuous RSA migration thresholds for acetabular cups. An association between early continuous migration and long-term aseptic loosening could not be determined. This finding is similar to that reported by Puijk et al. [17], which did not find an association between early continuous migration and long-term loosening of total knee arthroplasty (TKA) implants in a systematic review. For acetabular cups, it is possible that rotational movement may also need to be investigated, as rotational instability may contribute to implant loosening. In individual patients, a 2-year sagittal rotation of 2.53° in cemented acetabular cups was a strong risk factor for long-term aseptic loosening in primary THA [18]. Future studies should investigate thresholds for early sagittal rotation of acetabular cups to identify implants at risk of long-term loosening.

The thresholds established in this study are specifically intended to assess the risk of revision due to aseptic loosening of acetabular cups. Additionally, the thresholds should be interpreted in the context of the mean of RSA migration measurements of an appropriately sized cohort of primary THA cups [6]. The thresholds are not intended to be applied to an individual patient as different thresholds have been established for that purpose. Nieuwenhuijse et al. found that proximal migration greater than 1.76 mm was indicative of individual acetabular loosening in cemented acetabular designs in primary THA [18]. Kim et al. found that uncemented acetabular cups used at revision THA that migrated > 1 mm at 2 years correctly predicted individual implants to be loose at re-revision in 80% of cases [19]. Abrahams et al. reported that individual uncemented acetabular cups used at revision THA that migrated > 3 mm at any time point was diagnostic of loosening [20]. Klerken et al. reported that for individual acetabular cups in revision THA, for every 1 mm of proximal migration at 2 years postoperatively there was a 37% increased risk of aseptic loosening [21].

### Strengths

A strength of our study was that the 1- and 2-year migration thresholds were based on long-term revision rates from 2 international registries, ensuring that the thresholds are derived from the largest possible patient population. Arthroplasty registries capture the majority of joint replacements performed within a country and provide a comprehensive overview of the clinical outcomes across all patient populations, surgical techniques, fixation techniques, and implant designs [22,23]. In addition to registry data, this study included the implant-survival combinations from the previously published threshold study [4] to strengthen the analysis. Whilst registry data serves as the standard for long-term implant performance, the previously published migration threshold used cohort survivorship studies. By including this data, we were able to include acetabular cups introduced prior to the introduction of the AOANJRR and LROI, in 1999 and 2007, respectively. Incorporating historic implant data that has demonstrated high early RSA migration and unacceptable long-term revision rates remains important when establishing surrogate benchmarks against which new implant performance can be assessed.

### Limitations

First, the established 1- and 2-year migration thresholds represent an association between early proximal RSA migration and long-term revision rates due to aseptic loosening. However, these early migration thresholds do not imply causation for long-term loosening and do not account for overall implant survivorship beyond aseptic loosening. As RSA studies measure only implant migration, they are not suited to investigate other failure mechanisms such as periprosthetic fracture, dislocation, or infection. Second, some of the acetabular cup designs that were identified in the RSA review did not have corresponding registry revision rates. Newer cup designs studied with RSA may not have 10-year survivorship data, or they may not have been used widely across the 2 international registries (LROI and AOANJRR). This raises questions concerning external validity; however, there were 164 different implant-survival combinations in this study from the 2 registries. Third, this study did not establish fixation-specific acetabular migration thresholds. For example, this study did not create a separate threshold for cemented and uncemented acetabular cups, cups with screw or screwless fixation, or for cups matched with highly cross-linked polyethylene or conventional polyethylene liners. In this respect, a previous meta-analysis found that implant factors did not significantly influence early acetabular cup migration [8]. Additionally, creating fixation-specific thresholds was not feasible as there were not enough uncemented acetabular implant designs that had a high level of early migration. However, the present study matched the survivorship data from the national registries for the implant and its respective fixation techniques as identified from the RSA studies. This study provided a large dataset and allowed the matching of the acetabular cups to be as accurate as possible to the descriptions provided from the RSA studies at an implant fixation level. Nonetheless, the migration patterns in the review were not investigated beyond 10 years [8], and fixation may influence migration patterns at longer follow-ups, such as 15 or 20 years. Finally, this study did not update the 2012 literature review to identify new published cohort reports of acetabular cup survivorship, as publication bias may reduce the chances of poorly performing implants being published. However, in our study, we used the national registry survivorship data, which is considered the highest quality evidence, as it captures the survivorship of all acetabular cups in the country.

### Conclusion

We confirmed the existing 2-year migration thresholds and established a new threshold at 1 year. We found no association between continuous migration between 1 and 2 years and 10-year revision rates. The 1-year migration threshold is the earliest surrogate measure for assessing late loosening of the acetabular cup and may identify underperforming implants.

*In perspective*, the established thresholds should not be the sole method for evaluating early implant safety but rather used in conjunction with other implant surveillance methods. The 1-year migration threshold will assist implant manufacturers and researchers to shorten assessment periods and reduce the number of patients at risk of receiving underperforming implants.

## Notes

Conceptualization: CHC, JA, BP, SC. Methodology: CHC, BP, SC. Investigation: CHC, JA, BP, SC. Validation: CHC, SC. Formal analysis: CHC. Data curation: CHC, BP, CH, LVS. Writing original draft: CHC. Writing review and editing: CHC, SC, JA, LBS, TR, CW, CH, LVS, BP. Visualization: CHC, JA, SC. Supervision: JA, BP, SC. Project administration: CHC, JA, BP.

Handling co-editors: Keijo Mäkelä and Robin Christensen

*Acta* thanks Maziar Mohaddes and Stephan Maximilian Röhrl for help with peer review of this manuscript.

## References

[1] Dunbar M, Ryd L. The power of registries and radiostereometric analysis (RSA). Acta Orthop 2025; 96: 11-12. doi: 10.2340/17453674.2024.41169. 39776208 PMC11706015

[2] Nelissen R G, Pijls B G, Kärrholm J, Malchau H, Nieuwenhuijse M J, Valstar E R. RSA and registries: the quest for phased introduction of new implants. J Bone Joint Surg Am 2011; 93(Suppl 3): 62-5. doi: 10.2106/JBJS.K.00907. 22262426

[3] Pijls B G, Nelissen R G. The era of phased introduction of new implants. Bone Joint Res 2016; 5(6): 215-17. doi: 10.1302/2046-3758.56.2000653. 27267796 PMC4921053

[4] Pijls B G, Nieuwenhuijse M J, Fiocco M, Plevier JW , Middeldorp S, Nelissen R G. Early proximal migration of cups is associated with late revision in THA: a systematic review and meta-analysis of 26 RSA studies and 49 survival studies. Acta Orthop 2012; 83(6): 583-91. doi: 10.3109/17453674.2012.745353. 23126575 PMC3555453

[5] Malchau H, Bragdon C R, Muratoglu O K. The stepwise introduction of innovation into orthopedic surgery: the next level of dilemmas. J Arthroplasty 2011; 26(6): 825-31. doi: 10.1016/j.arth.2010.08.007. 20888183

[6] Kaptein B L, Pijls B, Koster L, Karrholm J, Hull M, Niesen A. Guideline for RSA and CT-RSA implant migration measurements: an update of standardizations and recommendations. Acta Orthop 2024; 95: 256-67. doi: 10.2340/17453674.2024.40709. 38819193 PMC11141406

[7] Kärrholm J, Gill R H S, Valstar E R. The history and future of radiostereometric analysis. Clin Orthop Relat Res 2006; 448. doi: 10.1097/01.blo.0000224001.95141.fe. 16826090

[8] Cho C H, Pijls B G, Abrahams J M, Roerink A, Katembwe R, Baker A, et al. Migration patterns of acetabular cups: a systematic review and meta-analysis of RSA studies. Acta Orthop 2023; 94: 626-34. doi: 10.2340/17453674.2023.24580. 38157007 PMC10757199

[9] LROI. Dutch Arthroplasty Register. LROI; 2023. Available from: https://www.lroi.nl/

[10] AOANJRR. The Australian Orthopaedic Association National Joint Replacement Registry Adelaide South Australian Health and Medical Research Institute; 2023. Available from: https://aoanjrr.sahmri.com/

[11] EU regulation 2017/745 of the European Parliament and the Council of 5 April 2017 on medical devices. Official Journal of the European Union 2023. WEBLINK??

[12] Malak T T, Broomfield J A, Palmer A J, Hopewell S, Carr A, Brown C, et al. Surrogate markers of long-term outcome in primary total hip arthroplasty: a systematic review. Bone Joint Res 2016; 5(6): 206-14. doi: 10.1302/2046-3758.56.2000568. 27267795 PMC4921042

[13] Van de Vusse S F, De Laat N N, Koster L A, Kaptein B L. The accuracy and precision of CT-RSA in arthroplasty: a systematic review and meta-analysis. Acta Orthop 2025; 96: 295-303. doi: 10.2340/17453674.2025.43334. 40159987 PMC11971844

[14] Ryd L, Albrektsson B E, Carlsson L, Dansgård F, Herberts P, Lindstrand A, et al. Roentgen stereophotogrammetric analysis as a predictor of mechanical loosening of knee prostheses. J Bone Joint Surg Br 1995; 77(3): 377-83. doi: 10.1302/0301-620X.77B3.7744919. 7744919

[15] Kärrholm J, Borssén B, Löwenhielm G, Snorrason F. Does early micromotion of femoral stem prostheses matter? 4–7-year stereoradiographic follow-up of 84 cemented prostheses. J Bone Joint Surg Br 1994; 76(6): 912-17 .doi: 10.1302/0301-620X.76B6.7983118. 7983118

[16] Derbyshire B, Prescott R J, Porter M L. Notes on the use and interpretation of radiostereometric analysis. Acta Orthop 2009; 80(1): 124-30. doi: 10.1080/17453670902807474. 19234894 PMC2823227

[17] Puijk R, Singh J, Puijk R H, Laende E K, Plevier J W M, Nolte P A, et al. Evaluation and refinement of thresholds for early migration of total knee replacements as an estimator of late aseptic loosening: an updated systematic review of RSA and survival studies. Acta Orthop 2025; 96: 1-10. doi: 10.2340/17453674.2024.42574. 39776207 PMC11706017

[18] Nieuwenhuijse M J, Valstar E R, Kaptein B L, Nelissen R G. Good diagnostic performance of early migration as a predictor of late aseptic loosening of acetabular cups: results from ten years of follow-up with Roentgen stereophotogrammetric analysis (RSA). J Bone Joint Surg Am 2012; 94(10): 874-80. doi: 10.2106/JBJS.K.00305. 22617914

[19] Kim Y S, Abrahams J M, Callary S A, De Ieso C, Costi K, Howie D W. Proximal translation of > 1 mm within the first two years of revision total hip arthroplasty correctly predicts whether or not an acetabular component is loose in 80% of cases: a case-control study with confirmed intra-operative outcomes. Bone Joint J 2017; 99-B(4): 465-74. doi: 10.1302/0301-620X.99B4.BJJ-2016-0805.R1. 28385935

[20] Abrahams J M, Kim Y S, Callary S A, De Ieso C, Costi K, Howie D W. The diagnostic performance of radiographic criteria to detect aseptic acetabular component loosening after revision total hip arthroplasty. Bone Joint J 2017; 99-B(4): 458-64. doi: 10.1302/0301-620X.99B4.BJJ-2016-0804.R1. 28385934

[21] Klerken T, Mohaddes M, Nemes S, Kärrholm J. High early migration of the revised acetabular component is a predictor of late cup loosening: 312 cup revisions followed with radiostereometric analysis for 2–20 years. Hip Int 2015; 25(5): 471-6. doi: 10.5301/hipint.5000246. 25952912

[22] Zhou Y, Wall C J, Stevens J, Fraval A, Lewis P L, McAuliffe M J. Data resource profile: the Australian Orthopaedic Association National Joint Replacement Registry (AOANJRR). Int J Epidemiol 2025; 54(4): dyaf078. doi: 10.1093/ije/dyaf078. 40524466 PMC12199916

[23] LROI. Completeness. The Dutch Arthroplasty Register; 2025. Available from: https://www.lroi.nl/jaarrapportage/data-quality/completeness/#Completeness-per-year

